# Prediction of Low-Dose Aspirin-Induced Gastric Toxicity Using Nuclear Magnetic Resonance Spectroscopy-Based Pharmacometabolomics in Rats

**DOI:** 10.3390/molecules27072126

**Published:** 2022-03-25

**Authors:** Abubakar Sha’aban, Hadzliana Zainal, Nor Azlina Khalil, Fatimatuzzahra’ Abd Aziz, Ewe Seng Ch’ng, Chin-Hoe Teh, Mustapha Mohammed, Baharudin Ibrahim

**Affiliations:** 1 School of Pharmaceutical Sciences, Universiti Sains Malaysia, Penang 11800, Malaysia; faa@usm.my (F.A.A.); mohammedmmrx@gmail.com (M.M.); 2 Department of Clinical Pharmacy and Pharmacy Practice, Faculty of Pharmaceutical Sciences, Ahmadu Bello University, Zaria 810107, Nigeria; 3 Advanced Medical and Dental Institute, Universiti Sains Malaysia, Penang 13200, Malaysia; norazlinakhalil@usm.my (N.A.K.); eschng@usm.my (E.S.C.); 4 School of Medical and Life Sciences, Sunway University, Bandar Sunway 47500, Malaysia; 5 Bruker (Malaysia) Sdn Bhd, Bayan Lepas 11900, Malaysia; chin_hoe.teh@bruker.com; 6 Faculty of Pharmacy, Universiti Malaya, Kuala Lumpur 50603, Malaysia

**Keywords:** aspirin, pharmacometabolomic, nuclear magnetic resonance, spectroscopy, gastric toxicity, multivariate analysis

## Abstract

Background: Low-dose aspirin (LDA) is the backbone for secondary prevention of coronary artery disease, although limited by gastric toxicity. This study aimed to identify novel metabolites that could predict LDA-induced gastric toxicity using pharmacometabolomics. Methods: Pre-dosed urine samples were collected from male Sprague-Dawley rats. The rats were treated with either LDA (10 mg/kg) or 1% methylcellulose (10 mL/kg) per oral for 28 days. The rats’ stomachs were examined for gastric toxicity using a stereomicroscope. The urine samples were analyzed using a proton nuclear magnetic resonance spectroscopy. Metabolites were systematically identified by exploring established databases and multivariate analyses to determine the spectral pattern of metabolites related to LDA-induced gastric toxicity. Results: Treatment with LDA resulted in gastric toxicity in 20/32 rats (62.5%). The orthogonal projections to latent structures discriminant analysis (OPLS-DA) model displayed a goodness-of-fit (R^2^Y) value of 0.947, suggesting near-perfect reproducibility and a goodness-of-prediction (Q^2^Y) of −0.185 with perfect sensitivity, specificity and accuracy (100%). Furthermore, the area under the receiver operating characteristic (AUROC) displayed was 1. The final OPLS-DA model had an R^2^Y value of 0.726 and Q^2^Y of 0.142 with sensitivity (100%), specificity (95.0%) and accuracy (96.9%). Citrate, hippurate, methylamine, trimethylamine N-oxide and alpha-keto-glutarate were identified as the possible metabolites implicated in the LDA-induced gastric toxicity. Conclusion: The study identified metabolic signatures that correlated with the development of a low-dose Aspirin-induced gastric toxicity in rats. This pharmacometabolomic approach could further be validated to predict LDA-induced gastric toxicity in patients with coronary artery disease.

## 1. Introduction

Coronary artery disease (CAD) is a leading cause of cardiovascular disease (CVD) related morbidity and mortality globally [1]. Low-dose aspirin (LDA) is the mainstay for the secondary prevention of CAD [2]. Aspirin inhibits platelet activity by irreversibly deactivating cyclooxygenase-I (COX-1), leading to the inhibition of platelet thromboxane-A2 (TXA2) production and TXA2-mediated platelet activation [3]. The activity of Aspirin on TXA2 explains its distinct efficacy in preventing atherothrombosis and shared gastrointestinal (GI) side effects with other antiplatelets [3]. Alternative antiplatelets or co-administration with gastro-protective agents are presently the most common strategies to reduce Aspirin-induced GI side effects [4,5]. However, alternative Aspirin use is limited with cost burden, pill burden and decreased effectiveness, necessitating the need for more cost-effective strategies.

There are limited studies on strategies, such as pharmacometabonomics, that predict the manifestation of gastric toxicity prior to LDA dosing. Pharmacometabolomics is a fast, economical and less invasive approach to predict drug-induced toxicity and complements personalized therapy. Proton nuclear magnetic resonance (^1^H-NMR) spectroscopy is a relatively new methodology for predicting drug effects using pre-dosed biomarkers of biofluids. NMR spectroscopy-based pharmacometabolomics is defined as “the prediction of the outcome (e.g., toxicity or efficacy) of a drug or xenobiotic in individuals, based on a mathematical model of pre-intervention metabolite signatures” [6]. NMR spectroscopy is the “gold standard” in pharmacometabolomics because of its non-destructive nature and enables the observation of the dynamics, partition and the quantification of metabolites in bio-samples. The pharmacometabolomics combines ^1^H-NMR and multivariate analysis in order to provide a detailed examination of the changes in the metabolic signatures of bio-samples. Therefore, this study aimed to identify metabolites that predict LDA-induced gastric toxicity using ^1^H-NMR-based pharmacometabonomics in rats.

## 2. Results

### 2.1. Gastric Toxicity

**Figure 1:**
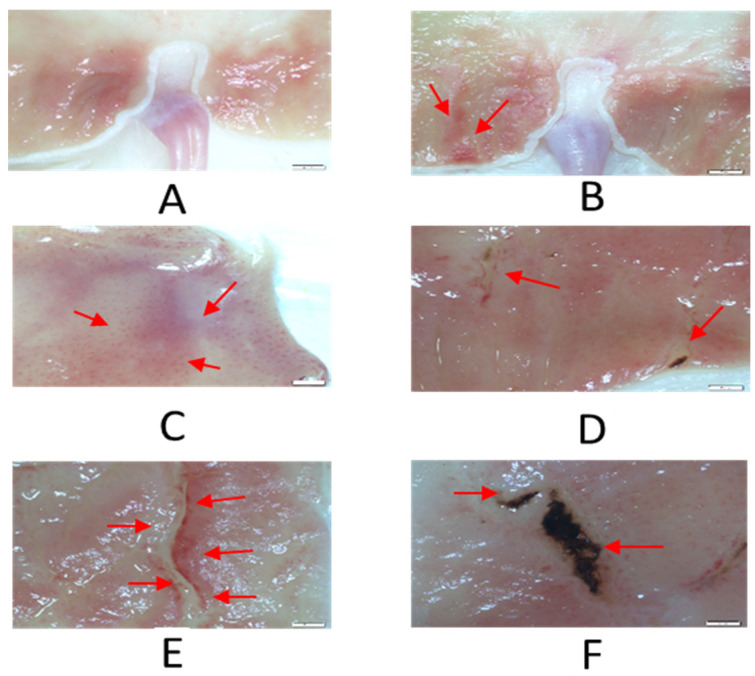
Different presentations of the gastric toxicity in rat stomach displayed by LDA. (**A**) No lesion, (**B**) red streaks, (**C**) oetechiae, (**D**) small lesions, (**E**) ulceration and (**F**) haemorrhagic ulceration. Magnification: 6.7×.

At the end of the low dose aspirin dosing period (28 days), none of the rats 0/10 (0%) in the vehicle-treated group had any form of gastric toxicity. However, most rats 20/32 (62.5%) in the LDA-treated group developed gastric toxicity. Representative samples of the gastric toxicity are shown in Figure 1.

### 2.2. Pre-Dose Profiling Models

**Figure 2:**
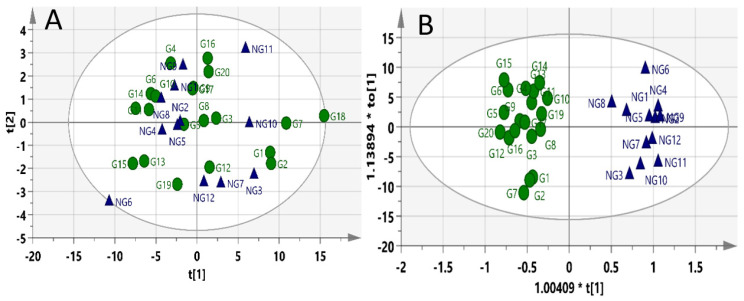
Score plots for the profiling model: (**A**) PCA and (**B**) OPLSDA. Blue triangles = non-gastric toxic rats; green circles = gastric toxic rats.

Principal Component Analysis (PCA) did not show clear discriminations between the groups. However, the Orthogonal Projections to Latent Structures Discriminant Analysis (OPLS-DA) score plots for the profiling model displayed clear discrimination between gastric toxic and non-toxic groups, as shown in Figure 2. The model had a goodness-of-fit value (R^2^Y) of 0.947 (very close to 1). However, the goodness-of-prediction value (Q^2^Y) of −0.185 (<0.4) indicates that the model has a poor predictive capacity.

**Figure 3:**
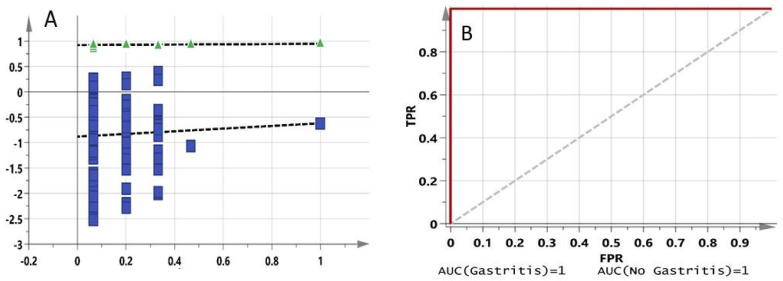
(**A**) Permutation plot for pre-dose rat urine profiling model. (**B**) AUROC curve for pre-dose rat urine profiling model.

The model has perfect (100%) sensitivity, specificity and accuracy values. It also has a perfect AUROC curve value of 1. The permutation test provided an R^2^Y intercept value of 0.919 and a Q^2^Y intercept value of −1.02 (Figure 3A). There is an overlap between the red and blue lines of the AUROC curve because the AUC value is 1 in both cases (Figure 3B).

The number of variables with VIP value >1 was 72, as highlighted in Table 1. Further examination and exclusion of spectral noise using Topsin resulted in 10 regions ascertained as signals integrated with Topspin before uploading to SIMCA for further screening and identifying useful discriminating metabolites.

### 2.3. Pre-Dosed Identification Models

**Figure 4:**
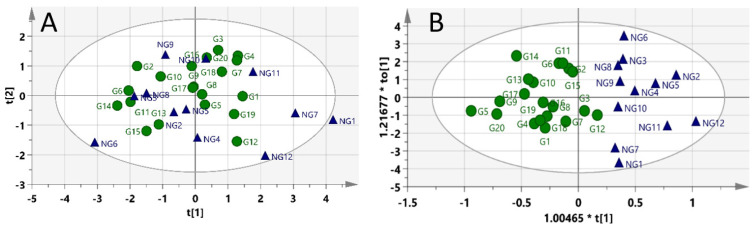
Score plots of pre-dose rat urine identification model: (**A**) PCA and (**B**) OPLSDA. Blue triangles = non-gastric toxic rats; green circles = gastric toxic rats.

PCA did not show clear discrimination between the two groups in Figure 4A. Except for two gastric toxic samples (G12 and G3) misclassified to be in the non-gastric toxic group, the pre-dose urine OPLSDA model successfully segregated the samples into gastric toxic and non-toxic groups, as shown in Figure 4B. The goodness values are also summarized in Table 1. R^2^Y value was 0.726, and the Q^2^Y value was 0.142. The sensitivity, specificity and accuracy of the identification model were 100%, 95% and 96.88%, respectively.

**Figure 5:**
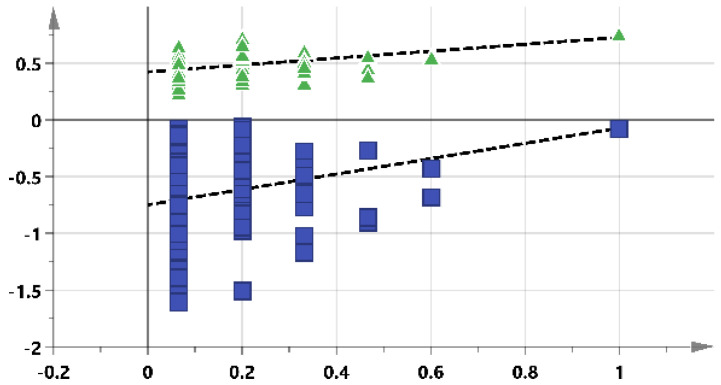
Permutation plot for pre-dose rat urine identification model.

The model also has an AUROC value of 1. Furthermore, the model was internally validated using the permutation plot (Figure 5). The pre-dose rat urine identification model passed almost all validity criteria plots. The majority of the Q^2^ values to the left are lower than the original Q^2^ point on the right. The blue regression line of the Q^2^ point intersects the vertical axis (on the left) below 0 (−0.875). Moreover, most of the R^2^ values (to the left) are lower than the original R^2^ value.

### 2.4. Identification of Biomarkers to Predict LDA-Induced Gastric Toxicity

After searches in the three databases, the ten regions of the pre-dose rats’ urine were identified to correspond to five metabolites, as highlighted in Table 2. These five metabolites were identified as putative biomarkers that may predict LDA-induced gastric toxicity.

**Figure 6:**
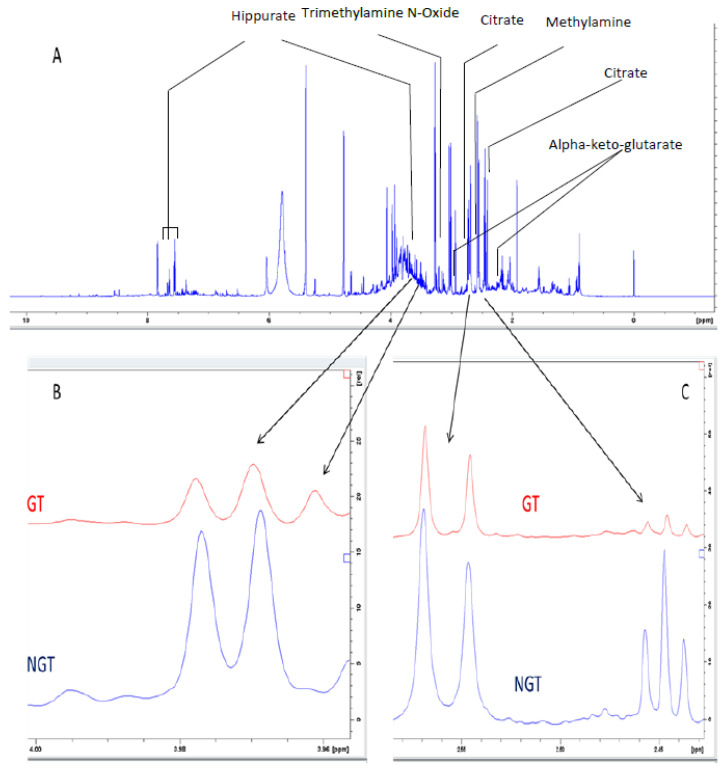
Example of spectral differences between gastric toxic (GT) and non-gastric toxic (NGT) rats. (**A**) The complete spectra. (**B**) Enlarged region showing 3.965–3.981 ppm. (**C**) Enlarged region showing 2.431–2.459 ppm and 2.531–2.571 ppm.

An example of the spectral differences in the metabolites identified between the gastric toxic and non-gastric toxic rats is depicted in Figure 6. The triplet at 2.431–2.459 ppm belonging to alpha-keto-glutarate, the doublet at 2.531–2.571 ppm belonging to citrate and a doublet at 3.965–3.981 ppm belonging to hippurate are used to show the differences.

## 3. Discussion

The study demonstrated the robustness of the model developed for pre-dosed prediction of LDA-induced gastric toxicity. The model reaffirms the assertion by Szymańska and colleagues that the area under the receiver operating characteristic (AUROC) or the number of misclassification is more precise in detecting biomarkers responsible for 2-class differentiation or discrimination [7]. They also proclaim that Q^2^ values are not very good as diagnostic statistics in discriminatory analysis models such as orthogonal projections to latent structures discriminant analysis (OPLS-DA) [7]. The goodness-of-fit (R^2^Y) value of 0.947 indicates the near-perfect reproducibility of the model. However, the goodness-of-prediction (Q^2^Y) value of −0.185 (less than the recommended 0.4 for biological models) shows that the model has poor predictive capacity [8]. The goodness-of-prediction values even lower than 0.3 have been reported in several metabolomic studies [9,10]. In such instances, it is recommended that the models are further assessed using permutation tests [10,11]. 

Although the AUROC value of 1 confirms the validity of the model, internal validation by conducting a permutation test indicates overfitting in the model. This may be due to the limited number of samples to perform an external validation of the model. Based on the aforementioned statistical parameters, this model may be suitable for predicting LDA-induced gastric toxicity when externally validated with independent data. Previous researchers stated that models with good sensitivity and specificity are suited for both screening and confirmation of disease [12]. Therefore, the diagnostic statistics qualify the model for developing a diagnostic kit, which can be used clinically to screen patients with the propensity to develop LDA-induced gastric toxicity when validated in a human study. Logically, a minimal number of discriminating metabolites will promote their clinical acceptance and utility. Reducing the crucial bins from 72 to 10 makes the model more clinically relevant. When validated with human data, the five identified metabolites (corresponding to the ten significant bins) from the final model may be used to develop a diagnostic kit that can be clinically useful.

The citric acid (citrate) is a weak acid that is formed endogenously in the tricarboxylic acid (TCA) cycle or consumed through some foods. The TCA cycle is also known as the citric acid cycle. NSAIDs have been found to cause the opening of "mitochondrial permeability transition pore", consequently leading to the uncoupling of oxidative phosphorylation, increasing the resting state respiration and disrupting the mitochondrial transmembrane potential. These NSAID-induced changes play a significant function in initiating tissue damage [13]. Takeuchi and colleagues [13] found no changes in serum citrate concentration after administering NSAIDs, including aspirin. They, however, found a decrease in citrate levels in stomach tissue extracts compared with controls. They, therefore, deduced two events to be associated with NSAID-induced gastric injury: hyperactivity of collagenase in the stomach and a decrease in levels of citrate (and other metabolites) as indicators of altered TCA cycle activity, which is a mitochondrial pathway. Other researchers [14] found citrate to be statistically reduced in the NSAID-induced gastric damage group compared to the control. Moreover, Takeuchi, et al. [15] reported no change in the serum levels of citrate after low-dose aspirin.

Methylamine is an endogenous metabolite resulting from the breakdown of amine. Its tissue level is found to increase in some disease conditions such as diabetes mellitus. The levels of methylamine and ammonia are mutually controlled by a multi-functional enzyme known as semicarbazide-sensitive amine oxidase (SSAO). The activity of SSAO deaminates methylamine to formaldehyde, thereby producing ammonia and hydrogen peroxide. An increase in serum SSAO activity has been reported in patients with some disease conditions, including diabetes mellitus, Alzheimer’s disease and vascular disorders. The deamination of methylamine catalyzed by SSAO results in the production of toxic formaldehyde. In this study, methylamine is one of the predictors of LDA-induced gastric toxicity in the urine. The exact mechanism/pathway linking aspirin-induced GI toxicity and methylamine has not been established. Perhaps future studies can focus on identifying the links that may show other pathways related to aspirin-induced GI toxicity.

Trimethylamine N-oxide (TMAO) results from the oxidation of trimethylamine. It is a common metabolite in both humans and animals. Specifically, TMAO is endogenously synthesized from trimethylamine derived from choline. Choline is usually obtained from either dietary lecithin or carnitine [16]. A link between blood and urinary levels of TMAO and gut microbiota has been established [17]. The concentration of TMAO increases if the number of bacteria that convert the trimethylamine to TMAO in the gut increases. It can, therefore, be inferred from this that subjects having a higher number of microbes that promote the synthesis of TMAO are also at an increased risk of developing aspirin-induced gastric toxicity. Previous researchers reported that NSAIDs increase the level of TMAO compared to controls [14].

Hippuric acid is a product of the conjugation of benzoic acid with glycine. It is referred to as an acyl glycine. Acyl glycines are synthesized through an enzyme known as glycine N-acyl transferase. Hippuric acid is a common constituent of urine, and its quantity is increased with an increase in the intake of phenolic compounds such as tea, fruit juices and wine. These phenols are changed to benzoic acid, which is subsequently converted to hippuric acid and excreted in urine. Gastrointestinal microflora appears to be responsible for quinic acid metabolism in hippuric acid. Indomethacin has been found to cause a decrease in hippurate, possibly due to the disruption of the normal microorganisms in the gastrointestinal tract [18].

One of two ketone derivatives of glutaric acid is alpha-ketoglutaric acid, also known as 2-oxoglutaric acid. When used without qualification, the word “ketoglutaric acid” usually always refers to the alpha version. The only difference between beta ketoglutaric acid and other ketoglutaric acids is the position of the ketone functional group, and it is significantly less prevalent. Alpha-ketoglutarate, commonly known as 2-oxoglutarate, is a biologically significant carboxylate. It is a keto acid formed when glutamate is deaminated, and it is an intermediate in the Krebs cycle [19]. 

## 4. Materials and Methods

### 4.1. Animals

Male Sprague-Dawley (SD) rats (250–300 g body weight) were obtained from the Animal Research Complex (ARC) of Advanced Medical and Dental Institute (AMDI), Universiti Sains Malaysia and acclimatized to the animal research room for seven days. The rats were given access to Altromin-1320 maintenance diet for rats/mice (Altromin International, Lage, Germany) and water ad libitum. The rats were housed in a room maintained at a temperature of 20 ± 2 °C, relative humidity of 55 ± 10%, respectively, and a 12 h light/dark cycle throughout the study. The rats were kept in individual cages (1 rat per cage) when the samples were not taken but placed in metabolic cages during the periods for sample collection. The experimental protocol was approved by the Institutional Animal Care and Use Committees (IACUC). All procedures were carried out according to the recommendations of IACUC.

### 4.2. Experimental Protocol

Two experimental groups were designed using a stratified randomization system based on rats’ bodyweight viz: group I (control, n = 10) and group II (treatment, n = 32). The rats in group I were administered 1% methylcellulose (10 mL/kg), while those in group II were administered low-dose aspirin, LDA (10 mg/kg) per oral for 28 days through the intra-gastric (IG) route with an oral gavage. Aspirin, sparingly soluble in water, was suspended in 1% methylcellulose before administration. The LDA (10 mg/kg) was equivalent to the clinical dose of 100 mg daily for adults [20,21], and it was used in a similar study to demonstrate LDA-induced gastric toxicity in rats [21].

### 4.3. Sample Collection

The rats were transferred to individual metabolic cages three days before urine collection to acclimatize before sample collection [22]. Twenty-four-hour urine samples were collected on day 1 (pre-dosed) and day 28 (post-dosed) using the metabolic cage urine collector containing a preservative (0.5 mL of 100 mg/mL solution of sodium azide (NaN_3_)) [6]. The amount of each urine sample received was recorded, transferred into a 15 mL falcon tube, centrifuged at 2500× *g* for 10 min at 4 °C to remove particles [23] and aliquoted into two 2 mL microcentrifuge tubes. The aliquot and the remaining bulk of urine were stored in a −80 °C freezer until analysis by the proton nuclear magnetic resonance (^1^H-NMR) spectroscopy. All rats were euthanized on the 28th day of the experiment with an overdose of a ketamine/xylazine (91.0/9.1 mg/mL) cocktail. 

### 4.4. Stomach Preparation

Four millilitres of 10% aqueous buffered formalin was instilled IG using oral gavage for in situ intraluminal fixations to preserve the integrity of the stomach tissue before opening up the abdominal cavity for stomach harvesting [24]. The stomachs were detached after five minutes in situ fixation and excised along the greater curvature. They are subsequently rinsed with cold saline and pinned on a polystyrene board with the mucosa facing upward to flatten the stomach. After that, the stomachs were dried using a manual blower. Drying was necessary to enhance sample visualization and prevent light reflection from the microscope. 

Each stomach sample was examined under the stereomicroscope (SZ61, Olympus Europa Holding GMBH, Hamburg, Germany). The software (Cellsens) was launched on a desktop monitor, and stomach images were captured. The image of the entire stomach could not be captured at once, even at the lowest zoom magnification (0.67×) and working distance (110 mm); hence, the segments were snapped and later stitched into a single image using Photoshop (Adobe Photoshop CS5 Version: 12.0), as recommended by the pathologist. The ulcerations and, likewise, the entire glandular stomach perimeter were measured with the aid of CellSens life science imaging software (Ver. 1.9 Olympus America, Inc., Center Valley, PA, USA). The stomachs were primarily classified based on the presence or absence of lesions (gastric toxicity).

### 4.5. Pharmacometabolomics Analysis

^1^H-NMR spectra were acquired at 700.14 MHz using an ASCEND™ 700MHz NMR Spectrophotometer (Bruker BioSpin Corp., Rheinstetten, Germany). A day preceding NMR analysis, aliquots of the urine samples to be analyzed were transferred from the freezer (−8 °C) into the refrigerator (4 °C) and allowed to thaw overnight (at least 8 h) to avoid sample degradation resulting from abrupt defrosting [25]. The thawed aliquots of the samples were centrifuged (MIKRO 22 R, HettichZentrifugen^®^, Tuttlingen, Germany) at 12,000× *g* at 4 °C for 5 min to remove any insoluble sediment from the solution. Urine measuring 400 µL and 200 µL of phosphate buffer were mixed (2:1) in a microcentrifuge tube [26] and vortexed for a few seconds to ensure uniform mixing. Then, 550 µL was transferred to a 5 mm NMR tube using a pipette and securely closed by its cap (BRUKER^®^, BioSpin, Rheinstetten, Germany). 

To ensure that an NMR tube was properly positioned, a sample gauge was used to align the NMR tube in the spinner. The tubes were then inserted into the respective sample holders and loaded into the spectrometer using Bruker’s IconNMR™ automation software. After the insertion of NMR tubes, the spectra of the ^1^H-NMR were acquired and processed using the automation interphase of IconNMR™ with TopSpin 3.5 (BRUKER^®^, BioSpin, Rheinstetten, Germany) software. The acquisition stages were locking, shimming and acquisition, and the data processing stages were Fourier Transform, phase correction and baseline correction. All spectra were acquired without spinning the sample. Each sample is given a lag time of five minutes (300 s) for thermal equilibration in the magnetic field before measuring 300 K. For each sample, the probe was automatically tuned and matched, and the magnetic field was locked on Urine+D2O and shimmed through a specifically optimized shim file for urine samples. Automatic ^1^H pulse calibration (pulsecal) was performed on each sample to reduce sample variability effects due to salt contents. ^1^H-NMR experiments were automated with the ICON NMR using the standardized acquisition and the processing parameters are as follows: pulse program (noesygppr1d), time domain (65536), dummy scans (4), scans (16), sweep width (20.5186 ppm), acquisition time (2.281 s), relaxation delay (4 s), receiver gain (12.70), dwell time (34.80 µs), mixing time (0.01 s), line broadening (0.30 Hz) and transmitter frequency offset (3289.90). The spectra were processed using Bruker Topspin 3.5 pl7.

### 4.6. Statistical Analysis

At the end of the experiments, the rats were classified into gastric toxic or non-gastric toxic based on the presence or absence of any gastric toxicity, respectively. Spectra were bucketed to 0.04 ppm using AMIX software (BRUKER^®^, Rheinstetten, Germany). Water (4.7–4.9 ppm) and urea (5.5–6.1 ppm) regions were excluded. The bucket table was then imported to SIMCA 14.1 software (MKS Umetrics^®^ Sweden, Umeå, Sweden). Skewed data were log-transformed. The data were then scaled using Pareto scaling (Pr scaling). 

Principal component analysis (PCA) was conducted, and the score plot was examined to explore the behaviour of the data. Hotelling’s T^2^ plot was used to discriminate intrinsic outliers in each group. The orthogonal partial least squares discriminant analysis (OPLSDA) was utilized to test the association between the buckets and gastric toxicity. This initial discriminatory model was the profiling model. The misclassification table was used to indicate the sensitivity, specificity and accuracy of each model. The best differentiating model was selected based on the two goodness values: the goodness-of-fit (R^2^Y) and goodness-of-prediction (Q^2^Y). A large R^2^Y (close to 1) is a necessary condition for a good model. It indicates good reproducibility. Likewise, a Q^2^Y value >0.5 signifies good predictivity. The variance between the two goodness values should not be too significant to ensure the right prediction and to avoid overfitting.

The variable importance for the projection (VIP) plot was then generated. The VIP plot summarizes the significance of the variables both to explain X (the predictors) and to correlate to Y (the outcome). The value of the VIP score, which is greater than 1, is the typical rule for selecting variables that are important, relevant and potentially discriminating [27,28]. Therefore, buckets with VIP value > 1 were chosen for further analysis. These spectral buckets (with VIP values > 1) were copied to an excel sheet and sorted in ascending order. The corresponding spectra for each bucket were verified in Topspin, and spectral noise was excluded from true signals. The 3-(trimethyl-silyl) propionic acid (TSP) peak was defined as the reference, and the peaks were calibrated by reference to its peak.

A new data table was created by copying the relative integrals with their corresponding chemical shift (ppm) into an Excel sheet. The TSP integral was excluded from the table to not affect the analysis (as it is only a reference) before importing to SIMCA. The data were also log-transformed, Pareto scaled and explored initially using PCA. OPLSDA was also applied, and VIP plots were generated. This second discriminatory model was the “Identification Model” (final model). The misclassification table was generated to show the proportion of correctly classified observations in the dataset. In SIMCA, the ability of a model to classify the individual subjects correctly or incorrectly is evaluated by the misclassification table tool [29,30].

Furthermore, the permutation plot (Y-scrambling) was used as an internal validation of the model. This compares the goodness tests (R^2^ and Q^2^) of the original model with the goodness test of several generated models by randomly permutating Y-observations (the outcome) while keeping the X-matrix (the predictors) constant. The number of permutations was set to 100 [31]. This means that the model was randomly built and validated 100 times. The AUROC curve was also computed to visualize the performance of the discriminatory models. It serves as a quantitative measure of the performance of the model. The performance parameter ranges between 0.5 (bad classification) and 1.0 (perfect classification).

### 4.7. Metabolites Identification

Metabolites were identified by systematically exploring three major databases, namely Biological Magnetic Resonance Data Bank (BMRB), Human Metabolome Database (HMDB) and Chenomx NMR Suite 6.0 (Chenomx^®^ Inc., Edmonton, AB, Canada). The quest begins by first exploring BMRB. The important chemical shifts identified from the identification model (previous step) were individually inputted in the designated field for exploring metabolites in the BMRB database. This generated several matching peaks along with their corresponding metabolites. The generated matching metabolites were individually cross-referenced in the HMDB database to ascertain their availability in the urine. The prospective metabolites available in the urine were further cross-matched in the Chenomx profiler to ascertain their identity.

## 5. Conclusions

The pharmacometabolomic analysis of the pre-dose ^1^H-NMR urine spectra identified metabolic signatures that correlated with the development of LDA-induced gastric toxicity and could predict gastric toxicity related to LDA. Citrate, hippurate, methylamine, trimethylamine N-oxide, and alpha-keto-glutarate were the putative metabolites identified and possibly implicated in LDA-induced gastric toxicity. The final model demonstrated good discriminatory properties, reproducibility and limited predictive capacity. This pharmacometabolomic approach can be translated to predict gastric toxicity in CAD patients when validated in humans.

## Figures and Tables

**Table 1:** Summary of pre-dose urine parameters for profiling and identification rat model.

	Number of Significant Metabolites (VIP > 1)	OPLSDA Score Plot Model		Multivariate Analysis		
		Goodness-of-Fit (R^2^Y)	Goodness-of-Prediction (Q^2^Y)	Sensitivity (%)	Specificity (%)	Accuracy (%)
Profiling model	72	0.947	−0.185	100	100	100
Identification model	10	0.726	0.142	95	100	96.88

**Table 2:** Potential pre-dose urine metabolites that may predict gastric toxicity.

Compound	Molecular Formula	Multiplicity	Chemical Shift
Citrate	C_6_H_5_O_7_	Doublet	2.677–2.718
		Doublet	2.531–2.571
Methylamine	CH_3_NH_2_	Singlet	2.680–2.700
Trimethylamine N-Oxide	C_3_H_9_NO	Singlet	3.277–3.284
Hippurate	C_9_H_9_NO_3_	Doublet	3.965–3.981
		Triplet	7.539–7.571
		Doublet	7.825–7.850
		Triplet	7.626–7.656
Alpha-keto-glutarate	C_5_H_6_O_5_	Triplet	2.431–2.459
		Triplet	2.996–3.024

## Data Availability

Not applicable.

## Sample Availability

Samples of the compounds low-dose Aspirin are available from the authors.
